# Treatment of the later stages of Parkinson’s disease – pharmacological approaches now and in the future

**DOI:** 10.1186/2047-9158-4-3

**Published:** 2015-02-12

**Authors:** Peter Jenner

**Affiliations:** Neurodegenerative Diseases Research Group, Institute of Pharmaceutical Sciences, Faculty of Health Sciences and Medicine, King’s College, London, SE1 1UL UK

## Abstract

The problems associated with the pharmacological treatment of the later stages of Parkinson’s disease (PD) remain those seen over many years. These centre on a loss of drug effect (‘wearing off’) with disease progression, the occurrence of dyskinesia, notably with L-dopa use and the appearance of non-motor symptoms that are largely refractory to dopaminergic medication. Treatment strategies in late PD have been dominated by the use of drug combinations and the subtle manipulation of drug dosage. However, change is occurring as the understanding of the basis of motor complications and fluctuations and non-motor symptoms improves. New pharmacological options are expanding with the advent of longer acting versions of existing dopaminergic drugs, new drug delivery systems and the introduction of non-dopaminergic agents able to manipulate motor function both within the basal ganglia and in other brain regions. Non-dopaminergic agents are also being investigated for the treatment of dyskinesia and for the relief of non-motor symptoms. However, while therapy continues to improve, the treatment of late stage PD remains problematic with non-motor symptoms dominating the unmet need in this patient group.

## Introduction

With disease progression and prolonged administration of dopaminergic medications, the treatment of later stage PD almost inevitably becomes more complicated [1, 2]. The need for dopamine replacement therapy becomes increasingly demanding as motor signs worsen. Those patients that were initially well controlled using dopamine agonists require the introduction of increasing amounts of L-dopa in the form of higher doses given at more frequent intervals. Those in whom therapy was started with L-dopa will likewise need the introduction of a longer acting dopamine agonist. However, it is the onset of motor fluctuations (‘wearing off’, ‘on-off’) and motor complications (dyskinesia, freezing) that bring the greatest difficulties in providing treatment that is adequate to maintain mobility of the quality seen in earlier disease. There is no predictability as to which patients will develop motor complications and fluctuations but the disease duration and stage, duration of exposure to L-dopa and dose, gender and body weight can all play a role [3, 4]. Once they have appeared, the difficulties associated with treating motor complications and motor fluctuations are a reflection of the incomplete understanding of their pathophysiology. Even if motor symptoms of PD are well controlled, numerous non-motor components of the illness will emerge that are more troublesome to the patient and that respond only partially, if at all, to dopaminergic medication [5]. The neuronal basis for non-motor symptoms is poorly understood and under researched leading to a situation where pharmacological approaches to their treatment are not obvious or not available. The difficulties encountered in treating the later stages of PD are a reflection of the widespread and progressive pathology of the disease process that characterises PD [6, 7]. It is in this area that we have strived to find disease modifying strategies through pharmacological means but so far failed [8].

This short review looks at some of the key areas of pharmacological intervention in later stage PD and examines how the current understanding of motor complications, motor fluctuations and non-motor symptoms has led to at least, some new approaches to the treatment of the later stage PD population. An emerging area is the use of non-dopaminergic drugs to treat both motor and non-motor symptoms of PD as well as the complications arising from treatment [9, 10]. The rationale behind this approach involves both the circuitry of the basal ganglia and those non-dopaminergic neurones affected by the pathology of PD. Within the basal ganglia, the dopaminergic input to the caudate-putamen (striatum) from zona compacta of substantia nigra is regulated at both the cell body and terminal level by numerous other neurotransmitters including glutamate, acetylcholine, 5-HT and noradrenaline and there are receptors for these transmitters located on dopaminergic neurones. For example, nicotinic receptors on dopaminergic terminals can regulate dopamine neuronal activity. Significant neuronal inputs from other brain stem nuclei, such as the raphe nuclei and locus coeruleus means in effect that these monoaminergic systems also play a key role in regulating basal ganglia function. The direct and indirect output pathways from the striatum are largely GABAergic in nature and provide inputs to the internal and external segments of the globus pallidus and to zona reticulate of substantia nigra [11]. Multiple other neurotransmitters affect the activity of these output neurones and they have acetylcholine, glutamate, 5-HT, noradrenaline, adenosine, opiate and cannabinoid receptors both on their cell bodies and terminals. All of these provide a potential pharmacological means of regulating motor function and the induction and control of dyskinesia. Notable is the large glutamatergic input from the cortex which completes the striatal-thalamic-cortical loop so essential for the control of voluntary movement. This pathway plays a key role in regulating the excitatory input to the basal ganglia and so has formed a key target in attempting to manipulate motor function. In a similar way, the GABAergic pathway from the external globus pallidus innervates the subthalamic nucleus (STN) which in turn sends a glutamatergic input to many areas of the basal ganglia, including substantia nigra. The key role of the STN controlling motor function has been shown by the effects of deep brain stimulation (DBS) in controlling dyskinesiaa and tremor in PD. Outside of the basal ganglia many non-dopaminergic neurones are progressively affected by pathology and biochemical change in PD [7]. These include the dorsal motor nucleus of the vagus, the raphe nuclei, the locus coeruleus, the pedunculopontine nucleus and the nucleus basalis of Meynert. The transmitters affected are acetylcholine, glutamate, 5-HT and noradrenaline among others and clearly these changes contribute to both motor and non-motor symptoms of PD and form novel targets for treating the disorder, in addition to dopaminergic medication.

### Dyskinesia – current and new treatment strategies

The occurrence of dyskinesia is still common in PD notably in those individuals treated with L-dopa [12]. However, disabling ‘troublesome’ dyskinesia does not appear to be as prevalent as in previous eras and this probably reflects the more cautious treatment of early PD with an emphasis on avoiding dyskinesia induction. The exception is those individuals with a young onset variant of PD who are highly vulnerable to the early occurrence of motor complications [4]. In contrast, ‘non-troublesome’ dyskinesia remains a frequent side-effect of L-dopa therapy in the PD population but often does not require treatment while other components of motor function are adequately maintained. A lessening of the impact of dyskinesia is also a reflection of the earlier detection and treatment of PD in general as the severity or duration of disease as judged by dopaminergic nigral cell loss, is a primary factor in determining the degree of exposure to L-dopa needed to initiate involuntary movements [13]. However, the impact of dyskinesia is in its inevitable expression by all forms of dopaminergic medication, once involuntary movements are established.

Previously, there has been a belief that holding L-dopa therapy back until required in later PD provides the same ‘honeymoon’ period for the control of motor symptoms as in early disease. Certainly, early use of dopamine agonists results in a lower incidence of dyskinesia in the short term [14]. But this approach may be incorrect as the progression of nigral cell death reduces the extent of L-dopa exposure and time required to prime the basal ganglia for the expression of involuntary movements [13]. Hence, the introduction of L-dopa in those with longer disease duration or in high doses, may result in a shortened period of good effect before motor complications appear [15–17]. Very recently, keeping the dose of L-dopa below 400 mg per day in early PD was shown to reduce the risk of dyskinesia induction [4]. Importantly, the early use of L-dopa was also shown to be the most effective treatment for motor symptoms and not to affect the long term risk of dyskinesia [18].

The underlying cause of dyskinesia is not known but it consists of two components – a persistent, if not permanent, priming process that lays down a motor memory for dyskinesia to appear every time dopaminergic treatment is administered – and the mechanism responsible for the expression of each episode of dyskinesia [19]. The nature of the priming process is poorly understood and no mechanism has been proposed that is not reversible over time unlike the motor memory for dyskinesia expression. Classically dyskinesia is attributed to changes in the direct output pathway from the striatum to the internal globus pallidus which has D-1 receptors on its cell bodies but this is not certain and the role of the indirect output pathway to external globus pallidus that has D-2 receptors on its cell bodies, has not been examined to the same extent. In fact, while changes in D-1 receptor signaling and transduction mechanisms accompany dyskinesia [20], pharmacological studies suggest that D-1 agonists are good anti-parkinsonian agents and induce no more dyskinesia than D-2 agonists once priming has occurred [21]. There is also debate about how alterations in presynaptic events can alter dopaminergic transmission so as to cause aberrant signaling at post-synaptic dopamine receptors and induce dyskinesia [22]. However, a more complicated explanation for dyskinesia expression would seem logical as it is a phenomenon that can be focal, segmental or generalised and involve chorea, dystonia and athetosis and so would seem unlikely to result from a single simple change in motor programming. However, a focus on basal ganglia output pathways has revealed numerous neuronal targets for the pharmacological manipulation of dyskinesia (see below) and these have been extensively explored in experimental models of PD with dyskinesia [23]. The two models most used are the unilateral 6-OHDA lesioned rat exposed to L-dopa and exhibiting abnormal involuntary movements (AIMs) and the MPTP treated primate with L-dopa induced dyskinesia. These are models that are highly predictive of the symptomatic effects of dopaminergic drugs in PD in man and where amantadine will suppress involuntary movements. However, their relevance as predictors of effect of non-dopaminergic medications remains in doubt since translation to man has been poor. This has been notably the case for non-dopaminergic drugs that have the twin objectives of improving or at least not worsening motor function and suppressing dyskinesia.

Common strategies for treating established dyskinesia involve a reduction in dopaminergic therapy or the use of formulations of dopaminergic drugs that reduce peak plasma levels as most dyskinesia is ‘peak dose’ in nature [24, 25] . However, both approaches can lead to a worsening of motor function where a fine balance between decreased mobility and the ‘troublesome’ nature of involuntary movements has to be addressed. The addition of amantadine can lessen dyskinesia in those individuals who can tolerate adequate doses of the drug as shown recently in a drug withdrawal study [26]. Amantadine has almost perfect pharmacokinetics with high oral bioavailability and a long plasma half-life [27] but many side-effects are ‘peak dose’ in nature and a result, a controlled release version of the drug is being evaluated in PD. The effects of amantadine are attributed to its actions as weak NMDA glutamate receptor antagonist – although this is not certain as it has multiple pharmacological actions. As a result a number of other glutamate antagonists have been evaluated for their effects on motor function in general and dyskinesia suppression in both man and in experimental models of PD [28] (Table 1). However, most glutamate receptor subtypes have a broad distribution in brain and identifying sub-types that have a more selective localisation in basal ganglia has been a challenge. NR2B antagonists have looked promising in experimental models of PD, notably the MPTP treated primate [29, 30], but not in man. AMPA antagonists such as talampanel and perampanel, again have appeared effective in preclinical testing but have been disappointing in clinical trial [31–33]. More recently antagonists or allosteric modulators of metabotropic glutamate receptors (mainly mGluR5), such as mavoglurant, have been investigated. At the doses used, these compounds appear to have some effect on dyskinesia with no significant worsening or improvement of motor disability [34, 35] but further investigation is required [36]. However, there appears to be a problem with the therapeutic window for glutamate antagonists in general that shows a very narrow dosage range in which the desired inhibition of dyskinesia without motor impairment occurs, below which no effect is observed but above which motor impairment becomes obvious and blockade of glutamate receptors in other brain areas starts to occur causing dizziness, ataxia, psychosis and vascular change [37]. So far, no new glutamate antagonists have succeeded in their clinical development for PD.

**Table 1:** **Examples of existing and novel approaches to the treatment of dyskinesia**

Drug class	Example	Status
*Glutamate antagonists*		
Non-specific NMDA	IR – amantadine	Marketed
	CR - amantadine	Clinical trials
NR2B		Experimental
AMPA	Talampanel, perampanel	Clinical trials
mGluR5	Mavoglurant	Clinical trials
*Other pharmacological classes*		
5-HT 1a/1b agonists	-	Experimental
5-HT 2c antagonists	-	Experimental
Adrenergic antagonists	Fipamezole	Experimental
Opioid agonists and antagonists	-	Experimental
Cannabinoid agonists	-	Experimental
Nicotinic agonists	-	Experimental
Anticonvulsants	Levetiracetam	Clinical trials

IR- immediate release; CR- controlled release.

Compounds of various other classes have also been investigated (Table 1) – including 5-HT agonists and antagonists, adrenergic antagonists, opiate agonists and antagonists, cannabinoid agonists and antagonists and anticonvulsants - and these have shown properties including extension of the duration of action of L-dopa and suppression of dyskinesia without motor impairment [10, 23, 38, 39]. Manipulating 5-HT function has seemed particularly promising and the use of 5-HT1a/1b agonists has proved highly effective in suppressing dyskinesia in experimental models [40]. Part of the rationale relates to the ability of 5-HT neurones to take up L-dopa and to convert it to dopamine that is then released and produces a non-physiological stimulation of striatal dopamine receptors [41]. But a more logical explanation relates to alteration in the serotoninergic input to the striatum from the raphe nuclei and the presence of multiple 5-HT receptor subtypes both on the terminals of this serotoninergic input and on the cell bodies of striatal output neurones. However, when taken in to clinical trial many non-dopaminergic drugs have not produced the predicted clinical effect. So, the face validity of the animal models for non-dopaminergic approaches needs re-evaluation and it may be that simple assessments of motor function in animals which can be disrupted through many means (sedation, hypotension, nausea, muscle weakness etc.) may not be a viable predictor of drug action outside of the dopaminergic arena. However, new avenues are currently being explored that utilise nicotinic agonists, highly selective 5-HT agonists, opiate agonists and antagonists and combinations of non-dopaminergic drugs of different classes.

But despite all this activity, dyskinesia remains an enigma – best avoided as once established, it is difficult to control and almost impossible to reverse. Continuous drug delivery provides an alternative approach to the treatment of dyskinesia and this will be considered later.

### ‘Wearing off’ – more troublesome than dyskinesia

Understanding the treatment of ‘wearing off’ is key to treating the later stages of PD as it eventually affects all patients and it is considered to have a bigger effect on quality of life by patient populations. ‘Wearing off’ should not be confused with the need for increased doses of dopaminergic medication due to disease progression. Rather ‘wearing off’ is reflected in the increasingly short duration of action of individual doses of L-dopa (and indeed, dopamine agonists) that occurs over time [42]. ‘Wearing off’ occurs earlier in the course of the progression of PD than most physicians (or patients) appreciate and significant numbers of patients develop ‘wearing off’ in the first few months or years after starting treatment [43, 44]. ‘Wearing off’ is characterised by a shorter sharper response to L-dopa that is not related to any change that occurs in the peripheral pharmacokinetics of L-dopa as reflected in plasma levels of the drug (although L-dopa absorption may become erratic). Rather, ‘wearing off’ is a phenomenon caused by alterations in the pharmacodynamics of L-dopa at the synaptic level. The classical explanation is that ‘wearing off’ is due to alterations in the presynaptic storage of L-dopa/dopamine in striatal dopaminergic neurones that buffers synaptic transmission against the fluctuations in plasma L-dopa levels occurring over the day in early PD [45]. As the disease progresses, this buffering capacity is lost and now the response to L-dopa more closely follows plasma drug concentrations leading to an increasingly short response. However, this cannot be the complete answer as post-synaptic changes must also contribute although how this occurs remains unknown but may reflect alterations in signal transduction and intra-cellular signaling mechanisms as a result of continuous post-synaptic dopamine receptor stimulation. This is based on the finding that ‘wearing off’ is seen on repeated administration of L-dopa to 6-OHDA lesioned rats and MPTP treated primates where the majority of dopaminergic terminals have been destroyed [46]. ‘Wearing off’ can also be seen in patients with PD treated with dopamine agonists that only act through post-synaptic receptor activation [47].

The treatment of ‘wearing off’ is based around providing more continuous delivery of dopaminergic medications and the more prolonged stimulation of post-synaptic dopamine receptors (Table 2). Classical approaches to the treatment of ‘wearing off’ involve increasing the dose and frequency of L-dopa administration, fractionation of L-dopa doses and multiple administrations, the introduction of sustained release forms of L-dopa, the use of rapid action forms of the drug through liquid suspensions or soluble pro-drug forms (eg. L-dopa methyl ester) [25, 48]. Renewed interest in L-dopa as first line therapy has led to the development of a number of new delivery forms, including gastro-retentive and sustained release formulations, which are currently in clinical development [49]. One recently approved formulation in the USA is IPX-066 (Rytary) which uses microspheres of different sizes to deliver L-dopa at different rates to provide a faster and more prolonged clinical response. In early and late stage patient populations, IPX-066 increases the daily duration of ‘on’ by approximately 3h compared to immediate release L-dopa and 1.5h compared to Stalevo [50, 51]. In the longer term, replacement of dopamine in the basal ganglia may be possible using gene therapy that inserts the key genes for dopamine production (tyrosine hydroxylase, dopa decarboxylase and GTP cyclohydrolase-1) in to the striatum. One viral vector based approach showing signs of success is ProSavin that has completed early stage clinical trial [52].

**Table 2:** **Existing and novel approaches to the treatment of ‘wearing off’**

Dopaminergic approaches	Examples	Status
*Drug combinations*		
L-dopa plus DA	IR – ropinirole. pramipexole	Marketed
DA plus L-dopa	IR – Sinemet, Madopar	Marketed
+ MAOB inhibitor	Irreversible – rasagiline, selegiline	Marketed
	Reversible - safinamide	Approved in EU
+ COMT inhibitor	Entacapone, tolcapone	Marketed
	Opicapone	Clinical trials
L-dopa + carbidopa + entacapone	Stalevo	Marketed
*Extended release*		
L-dopa	Sinemet CR	Marketed
	Rytary	Approved in USA
DA	ER – ropinirole, pramipexole	Marketed
	TD – rotigotine	Marketed
*Non-dopaminergic approaches*		
GABA/glutamate	Zonisamide	Marketed in Japan
A2a adenosine antagonist	Istradefylline	Marketed in Japan
*Gene therapy*		
TH/AADC/GTP-cyclohydrolase-1	ProSavin	Clinical trials

DA – dopamine agonist; IR- immediate release; CR – controlled release; TD – transdermal; TH-tyrosine hydroxylase; AADC – aromatic aminoacid decarboxylase.

Longer acting dopamine agonist are conventionally used to overcome the short duration of effect of L-dopa and transdermal delivery using rotigotine while once daily extended release oral formulations of pramipexole or ropinirole provide greater convenience [53, 54] (Table 2). The use of subcutaneous administration of apomorphine can relieve an unexpected ‘off’ [55], and other routes of immediate administration of apomorphine, such as the pulmonary, nasal or sublingual are under investigation.

Because L-dopa is proven to be the most effective drug for treating PD, the use of enzyme inhibitors (dopa decarboxylase inhibitors, COMT inhibitors and MAO-B inhibitors) to extend the duration of action of each dose of L-dopa or the dopamine derived from L-dopa, has proven another effective means of treating ‘wearing off’. The long acting COMT inhibitor tolcapone employed 3 times daily was an effective adjunct therapy for reducing ‘off’ time by blocking both peripheral and central COMT activity [56] but the potential for liver toxicity has limited its use [57]. Entacapone is shorter acting and only inhibits peripheral COMT and again is successfully employed to treat ‘wearing off’ [58]. It needs to be given with each dose of L-dopa either as a separate medication or in the triple combination of L-dopa, carbidopa and entacapone (Stalevo). New classes of COMT inhibitors, such as opicapone, are currently under development that have much longer plasma half-lives than entacapone and may only require once daily dosing [59]. MAO-B inhibitors can also be used to increase ‘on’ time in the later stage of PD. Selegiline and rasagiline are irreversible inhibitors of MAO-B so their effects are maintained for long periods of time with only once or twice daily dosing being required. Rasagiline is probably used more commonly than selegiline in most countries as it is ‘newer’ and has more recent clinical trials (PRESTO, LARGO) that show its effectiveness in treating ‘wearing off’ [60, 61]. Recently, a novel reversible selective MAO-B inhibitor safinamide was also shown to be effective in decreasing ‘off’ time in PD [62, 63] and this compound may soon be introduced in to the market place. Its reversibility would mean that any side-effects that arose from the inhibition of MAO could be rapidly reversed by stopping drug intake unlike the irreversible inhibitors where it would take many days for drug effect to disappear. Safinamide also possessesion channel blocking activity and these properties may convey additional therapeutic activity, including control of dyskinesia and improvements in cognitive function.

The emphasis on the treatment of ‘wearing off’ has remained centered on modifying dopaminergic medication. An exception is the use of A2a adenosine antagonists, notably istradefylline [64, 65]. Adenosine A2a receptors are selectively localised to the cell bodies and terminals of the indirect striatal output pathway so controlling a key pathway involved in motor dysfunction in PD. In clinical trials in PD in advanced fluctuating patients on optimal dopaminergic therapy, istradefylline can decrease ‘off’ time without an increase in ‘troublesome’ (as compared to non-troublesome) dyskinesia [66]. This has not been a consistent finding across all phase III studies reflecting the difficulties of the trial design and the extent of the placebo response that occurs in PD. However, in recent Japanese phase II/III investigations, a significant improvement in motor performance was demonstrated leading to the approval of istradefylline as a first in class adenosine antagonist for treating PD [67, 68]. Further clinical investigations are to undertaken in the USA/Europe and so istradefylline may become more widely available. A cautionary note would be that other adenosine A2a antagonists in clinical development for PD have failed for a variety of reasons despite good preclinical results and early evidence of efficacy (Factor et al., 2013; Hauser et al., 2011; Hodgson et al., 2009, most notably preladenant, but the reasons for a variable clinical effect are not clear [69–71]. In Japan, another non-dopaminergic drug, zonisamide, usually employed as an anti-epileptic agent, has found use in the treatment of ‘wearing off’ [72]. It has a complex mechanism of action that involves GABA and glutamatergic actions among others and it is uncertain how it produces its beneficial effects [73, 74] (Table 2).

### Pharmacological alternatives to oral therapy

Overall, the motor symptoms of PD are well controlled by dopaminergic medications in the early and middle stages of the illness. But oral dopaminergic medication may not provide the efficacy required as motor fluctuations and motor complications appear in later stage patients. Increasingly pharmacological treatment of these individuals has focused on the availability of drug delivery technologies that allow more continuous symptom relief. Strategies aimed at delivering both L-dopa and dopamine agonists (rotigotine, ropinirole, bromocriptine) by alternative routes (transdermal, pulmonary, buccal, nasal) are either already available or under development (Table 3). However, there are two approaches based on non-oral drug delivery, which have already shown their clinical utility, namely the infusion of apomorphine and L-dopa [75].

**Table 3:** **Examples of existing and novel non-oral dopaminergic delivery forms**

Non-oral therapies	Examples	Status
*L-dopa*		
Intraduodenal infusion	DuoDopa	Marketed
Subcutaneous infusion	ND0612L	Clinical trials
Inhalation	CVT-301	Clinical trials
*Apomorphine*		
Subcutaneous injection	APO-go pen	Marketed
Subcutaneous infusion	APO-go PFS	Marketed
Inhalation	-	Clinical trials
Intranasal	-	Clinical trials
Sublingual	APL-130277	Clinical trials
*DA agonists*		
Rotigotine transdermal	NeuPro	Marketed
Rotigotine subcutaneous depot	LY03003	Clinical trials
Bromocriptine intranasal	-	Experimental

Apomorphine is used as a subcutaneous injection for rescue therapy for unexpected ‘off’ periods but its major role has become in the treatment of unacceptable fluctuations and ‘off’ periods through subcutaneous infusion using programmable, high technology pumps. Infusions of apomorphine, in conjunction with oral dopaminergic therapy, improve motor symptoms and reducing ‘off’ time in later stage PD [76]. Over the longer term, continuous infusion of apomorphine may also reduce the severity of existing dyskinesia in some individuals [77]. The continuous drug delivery of apomorphine can reduce early morning akinesia and dystonia while allowing a reduction in the amount of oral dopaminergic medication required. While invasive and not without side-effects, this approach to treatment can lead to a substantive improvement in quality of life for those who are able to tolerate the drug [78].

The intra-duodenal infusion of L-dopa or its methyl ester, overcomes the variable and unpredictable effects of oral L-dopa administration by providing constant delivery of the drug to its site of absorption in the upper small intestine. By maintaining constant plasma levels of the drug over the course of the infusion, there are significant reductions in ‘off’ time in later stage PD [79–82]. This approach is again coupled to a longer term potential for a decrease in the severity of existing dyskinesia. The more continuous delivery of L-dopa would on theoretical grounds be a means of avoiding longer term motor complications and fluctuations by providing continuous dopaminergic stimulation but this concept needs to be tested. As with all invasive treatments and the problems associated with infusion technology, L-dopa infusion (DuoDopa) use is limited to a relatively small patient population but in those where it is effective, it can significantly improve the quality of life although peripheral neuropathy may be a greater risk than with oral L-dopa therapy [83].

### Treatment of non-motor symptoms

The treatment of PD to date has focussed heavily on the control of motor signs and the use of dopaminergic medications. However, non-motor symptoms affecting multiple body systems have now becoming accepted as part of PD [84, 85]. Non-motor signs can precede the onset of motor abnormalities but can also occur at the same time or following diagnosis of PD based on classical terminology. Later stage patients exhibit between 6–10 non-motor symptoms of PD and this represents major clinical challenge to physicians and a major determinant of disease outcome. Indeed, subtypes of PD may exist in which specific non-motor problems, such as neuropsychiatric disturbance, occur along with motor impairment [86]. While some non-motor symptoms show some response to dopaminergic therapy and get worse during ‘off’ periods [87], many are not significantly improved by dopaminergic medication. Non-motor symptoms are already treated with a variety of non-dopaminergic agents on an ‘as needs’ basis [2] (Table 4). For example, muscarinic antagonists are used for bladder dysfunction and excessive salivation, benzodiazepine derivatives and newer medications are used to induce sleep and modafinil in attempts to control day time somnolence. SSRI’s (paroxetine) and SNRI’s (venlafaxine) are used for depression although tricyclic antidepressants (amitriptyline, nortriptyline and desimpramine) may be more effective but possess anticholinergic activity [88]. Atypical antipsychotics, such as quetiapine, can improve psychosis and potentially, clozapine although its toxicity limits usage. Very recently, a 5-HT2a antagonist drug, primavanserin, was shown to effectively control psychosis in PD and this may eventually form a good addition to the available treatments [89]. The use of cholinesterase inhibitors, such as rivastigmine, to control the cognitive decline/dementia in later PD can have some effect but may potentially worsen tremor by altering the balance between dopaminergic and cholinergic function that exists in the basal ganglia. There seems to be less evidence of any beneficial effect of memantine in dementia in PD compared to AD or LBD [90]. One hope for PD is the development of multifunctional drugs for PD that combine MAO-B inhibitory activity with cholinesterase inhibitory properties but their efficacy needs to be proven [91].

**Table 4:** **Examples of existing drugs used to treat non-motor symptoms of PD**

Non-motor symptom	Example	Status
Bladder dysfunction	Anticholinergics - oxybutinin	Marketed
Depression/anxiety	SSRI - paroxetine	Marketed
	SNRI - venlafaxine	Marketed
	Tricyclic antidepressants – nortryptyline, desipamine	Marketed
	Dopamine agonist - pramipexole	Marketed
Psychosis	Atypical antipsychotics – quetiapine	Marketed
	5-HT antagonist - primavanserin	Approved in USA
Dementia	Cholinesterase inhibitors - rivastigmine	Marketed
Sleep disturbance - insomnia	Hypnotic - zolpidem	Marketed
Excessive daytime somnolence	Modafinil	Marketed

It could be argued that using drugs developed for similar indications outside of PD suffices for treating non-motor symptoms. In reality, what is required is drugs with multiple pharmacological actions that will treat multiple symptoms of the illness. But there is a lack of knowledge of the basis of non-motor symptoms and many arise from pathological change in non-dopaminergic brain regions outside of the basal ganglia. Overall, it is not known which brain regions and which neurotransmitter systems cause the onset of non-motor symptoms and how this relates to the spread of pathology in PD. Few systematic studies have been undertaken to match pathology in post-mortem brain tissue with carefully recorded non-motor symptomatology during life. Some imaging studies have been undertaken as the range of ligands available for looking at non-dopaminergic nuclei improves. For example, these have shown that degeneration of 5-HT fibres arising from the raphe nuclei is more prominent in those individuals with PD who have fatigue or depression as major non-motor symptoms [92, 93]. Non-motor symptoms of PD can also be seen in experimental models of PD, including the 6-OHDA lesioned rat and MPTP treated primate. These include sleep disturbance, cognitive, change and bladder dysfunction and some pharmacological analysis of potential treatments is starting to be undertaken [94–97]. Non-motor symptoms can also be seen in genetic models of PD in mice and these have recoded the presence of olfactory changes, changes in bladder and gut function and in neuropsychiatric signs, such as anxiety depression and cognition, although the results vary between models and little pharmacological manipulation has so far been reported [98, 99].

## Conclusions

This review has tried to set in place the current treatment of the later stage PD and the changing face of the approaches being used to improve the control of motor and non-motor symptoms. The problems faced by clinicians in the treatment of PD have changed with the increased survival rates and improvement in life expectancy of the patient population. Better control of motor symptoms in the long term has been achieved through the use of dopaminergic drugs in new formulations and delivery forms. A lower incidence of disabling dyskinesia is another consequence of more restrained early treatment with L-dopa and dopamine agonists. ‘Wearing off’ is being detected earlier and more effectively treated as a result of novel treatment strategies and new delivery forms of older drugs. Some non-dopaminergic medications are starting to appear most notably adenosine A2a antagonists but success in this area has not been as great as initially hoped. Now the challenge has become how to approach the treatment of non-motor symptoms of PD. The clinical phenomenology is well established and the relevance to long term outcomes in PD is clear. However, tailored treatment for non-motor symptoms of PD is still lacking and more basic research is needed to uncover potential therapeutic avenues. Finally, the issue that this short article did not address, namely how to achieve disease modification in PD and the possibilities for neurorestoration and cure, remains unsolved. As a consequence, we must continue to look at the long term treatment of patients with PD and be increasingly aware of the issues likely to occur in later stage patients and how these might best be treated.
